# Players over the Surface: Unraveling the Role of Exopolysaccharides in Zinc Biosorption by Fluorescent *Pseudomonas* Strain Psd

**DOI:** 10.3389/fmicb.2017.00284

**Published:** 2017-02-24

**Authors:** Anamika Upadhyay, Mandira Kochar, Manchikatla V. Rajam, Sheela Srivastava

**Affiliations:** ^1^Department of Genetics, University of Delhi South CampusNew Delhi, India; ^2^TERI Deakin Nanobiotechnology Centre, The Energy and Resources InstituteGurgaon, India

**Keywords:** biosorption, exopolysaccharides, alginates, biofilms, biocontrol

## Abstract

Fluorescent *Pseudomonas* strain Psd is a soil isolate, possessing multiple plant growth promoting (PGP) properties and biocontrol potential. In addition, the strain also possesses high Zn^2+^ biosorption capability. In this study, we have investigated the role exopolysaccharides (EPS) play in Zn^2+^ biosorption. We have identified that alginates are the prime components contributing to Zn^2+^ biosorption. Deletion of the *alg8* gene, which codes for a sub-unit of alginate polymerase, led to a significant reduction in EPS production by the organism. We have also demonstrated that the increased alginate production in response to Zn^2+^ exposure leads to improved biofilm formation by the strain. In the *alg8* deletion mutant, however, biofilm formation was severely compromised. Further, we have studied the functional implications of Zn^2+^ biosorption by *Pseudomonas* strain Psd by demonstrating the effect on the PGP and biocontrol potential of the strain.

## Introduction

Metals have become integral part of cellular functions, both structural and functional; and have been broadly classified as essential or non-essential metals. While the latter are extremely toxic, the former, including transition elements like Co^2+^, Mn^2+^, Ni^2+^, Fe^2+^, Cd^2+^, and Zn^2+^, can also exert the harmful effects beyond a certain concentration. To tackle this, bacterial systems have evolved a number of mechanisms to maintain a fine-tuned balance between metal deficiency and excess.

Zinc plays an important role as a trace element in organisms belonging to all domains of life, ranging from bacteria to humans, where it is present as the divalent cation (Zn^2+^; Blindauer, 2015). Zn^2+^ is unique among transition metals since it is redox inactive under physiological conditions (Mangold et al., 2013). It serves as the cofactor of a large number of the known enzymes (Choudhury and Srivastava, 2001; Blencowe and Morby, 2003; Blindauer, 2015) and is involved in DNA-protein interactions in the form of Zn^2+^-finger motifs (Matthews and Sunde, 2002). In the context of pathogens, Zn^2+^ is required for exhibiting full virulence by many pathogenic organisms (Shafeeq et al., 2013). This is the reason why Zn^2+^ availability is reduced during acute-phase response to a bacterial infection (Corbin et al., 2008; LeGrand and Alcock, 2012). Since Zn^2+^ is associated with a number of important cellular processes essential for growth and metabolism, its cellular levels need to be maintained in order to ensure proper availability of the metal for various processes, and at the same time preventing the cellular components from the deleterious effects of Zn^2+^ toxicity. Microbial communities, like all other organisms, have adapted themselves to the metal concentrations encountered by two major strategies, namely avoidance and sequestration. Based on these strategies, the major mechanisms operating in the prokaryotes that maintain cellular Zn^2+^ concentration can be classified into: (i) physico-chemical interactions (adsorption or biosorption to cell wall and other constituents); (ii) regulated import; (iii) efflux; and (iv) sequestration (Ledin, 2000; Choudhury and Srivastava, 2001; Blencowe and Morby, 2003; Upadhyay and Srivastava, 2014).

Biosorption of metals onto a microbial surface is a function of negatively charged cell surface and is dependent on the surface properties of the cell, such as charge and orientation of metal-binding functional groups, metal speciation and chemistry in aqueous phase (Ledin, 2000). Another important determinant of the biosorption process are the extracellular polymeric substances produced by bacteria, which confer an overall negative charge to the bacterial surface under circumneutral pH conditions, due to the presence of carboxylic and phosphoryl groups (Beveridge, 1988). The main constituents of extracellular polymeric substances include extracellular polysaccharides or exopolysaccharides (EPS), proteins, lipids, and DNA (Flemming and Wingender, 2001; Allesen-Holm et al., 2006). Bacterial EPS are implicated in a number of functions, such as adhesion to substratum, protection against anti-bacterial compounds and binding to organic molecules and inorganic ions (Ma et al., 2009). Additionally, bacterial EPS are also involved in metal adsorption due to the interaction between metal cations and negative functional groups of EPS (Ledin, 2000; Vijayaraghavan and Yun, 2008).

*Pseudomonas* sp. are known to secrete three major types of EPS, namely alginate, Psl and Pel Conti (Conti et al., 1994; Ma et al., 2009; Franklin et al., 2011; Ghafoor et al., 2011; Yang et al., 2011). Psl is a galactose and mannose-rich polysaccharide, aiding mainly in the initial attachment and mature biofilm formation (Ma et al., 2009). Generally produced during planktonic growth, this EPS mediates attachment to surfaces and formation of micro-colonies. The other polysaccharide, Pel, is a glucose-rich cellulose-like polymer required for pellicle formation at air-liquid interface (Friedman and Kolter, 2004). Alginates are linear EPS consisting of β-1,4-linked β-D-mannuronic acid and its C5 epimer α-L-guluronic acid (Remminghorst and Rehm, 2006). Produced only by two bacterial genera of *Pseudomonas* and *Azotobacter*, these EPS are responsible for a mucoid colony phenotype and are also the premier components of bacterial biofilms (Sutherland, 2001; Ghafoor et al., 2011; Whitfield et al., 2015). The alginate biosynthesis operon consists of 12 genes (*algD, alg8, alg44, algK, algE, algG, algX, algL, algI, algJ, algF, and algA*; Figure S1) under tight control of the promoter upstream of *algD* (Remminghorst and Rehm, 2006).

A number of pseudomonads, including *Pseudomonas aeruginosa, P. syringae, Pseudomonas putida*, and *Pseudomonas fluorescens*, serve as dominant members of Plant Growth Promoting (PGP) bacteria (Vessey, 2003; Couillerot et al., 2009; Bhattacharyya and Jha, 2012). In this context, EPS production and the biofilm formation is expected to play an important role in root colonization and also contribute to the rhizosphere competence of these bacteria. Fluorescent *Pseudomonas* strain Psd is a rhizosphere isolate, possessing multiple PGP properties and biocontrol potential (Upadhyay and Srivastava, 2008, 2010; Kochar et al., 2011). Besides, we have earlier demonstrated that this strain possesses high resistance toward Zn^2+^, which emanates from extracellular biosorption (Upadhyay and Srivastava, 2014). We have also established that Zn^2+^ biosorption is coupled with an increase in the total EPS production. In this communication, we dissect this aspect further and identify the key players that mediate the biosorption process. Since the strain in hand is a PGP bacterium, effect of Zn^2+^ biosorption and alginate production on the PGP potential of the strain is also demonstrated. These observations may provide important leads in ascertaining if the strain could continue to provide its beneficial effects even in soils contaminated with high levels of Zn^2+^ and be applied as a bioinoculant.

## Materials and methods

### Organism, culture conditions, and chemicals

Fluorescent *Pseudomonas* strain Psd, isolated from the roots of *Vigna mungo*, has been characterized as mentioned before (Upadhyay and Srivastava, 2008). The strain was maintained on Gluconate Minimal medium (GMM; Gilotra and Srivastava, 1997) with and without ZnSO_4_.7H_2_O. As per the experimental requirement, the media was supplemented with the appropriate concentration of autoclaved metal salt solution (ZnSO_4_.7H_2_O). The shake cultures were raised on Controlled Environment Shaker Incubator (Kühner, Switzerland) at 200 rpm at 30°C for the required period of time. Growth was determined turbidometrically at 600 nm, as per the protocol described earlier (Upadhyay and Srivastava, 2014). All chemicals used in the study were of analytical grade and purchased from Sigma Aldrich (St. Louis, MO, USA). All the other strains and plasmids used in this study along with their relevant characteristics are listed in Table S1.

### Isolation and purification of extracellular polysaccharides

EPS extraction was carried from 5 day-spent culture filtrate of strain Psd grown at different Zn^2+^ concentrations in GMM by the method described earlier (Bitton and Freihofer, 1978). Briefly, 2 volumes of 95% ethyl alcohol was added to the culture supernatant and was kept at 4°C overnight. The resulting precipitate was recovered by centrifugation at 8,000 × g for 10 min. The precipitate was dissolved in sterile distilled H_2_O and dialyzed at 4°C against distilled H_2_O for desalting. The purified EPS was concentrated under vacuum and stored at 4°C until use.

### Transmission electron microscopy

Cells grown at different Zn^2+^ concentrations were analyzed for the ultra-structural changes by Transmission Electron Microscopy (TEM). Pellets of sedimented cells, washed in saline, were fixed with 2.5% gluteraldehyde in 0.1 M phosphate buffer for 6 h at 4°C, and washed thrice with the same buffer. Dehydration was carried out in a graded ascending series of acetone followed by toluene. Specimens were infiltrated with 3:1 (v/v) mixture of toluene and Araldite [50% Epoxyresin + 50% Dodecenyl succinic anhydride (DDSA)] for 2 h, followed by pure Araldite, and were finally embedded in beam capsule with Araldite + Accelerator [(Trimethyl aminomethyl phenol (DMP-30)] and polymerized at 50°C for 24 h and 60°C for 48 h. Thin sections were cut with an Ultracut Microtome E (Ultracut E, Riechert Jung, Germany) and stained with uranyl acetate and lead citrate. The sections were analyzed in Transmission electron Microscope (FEI Electron Optics, USA).

### Fourier-transformed infrared spectroscopy

Fourier-Transformed Infrared (FT-IR) spectrum of purified EPS was recorded to elucidate the chemical binding environment of Zn^2+^. Analyses were performed on an FT-IR spectrophotometer (Tensor 37, Bruker Optics, USA), equipped with total attenuated reflectance (ATR) objective. The purified EPS from strain Psd grown at varying Zn^2+^ concentrations was scanned over a wave number range of 4,000–1,000 cm^−1^ with a resolution of 4 cm^−1^. Samples were placed over a Zn-Se crystal and the peak emanating from the crystal was obtained between wave number 2400–2200 cm^−1^. Background spectrum of water was collected and normalized prior to measurement of samples. For each sample, 16 scans were collected in order to evaluate the heterogeneity in the sample. All the spectra obtained were smoothened and their baseline was corrected.

## Expression analysis

### Total RNA extraction and cDNA preparation

Total RNA was isolated from bacterial cultures with the RNeasy Protect Bacteria Mini Kit for total RNA purification (Qiagen, Netherlands), as per manufacturer's instructions. Traces of DNA were removed from RNA preparations using DNase I, (Amplification Grade, Sigma Aldrich). DNase-free RNA (1 μg) was used in a one-step RT-PCR reaction (RevertAid First Strand cDNA Synthesis Kit, Thermo Scientific, USA) performed with the universal hexamer primers provided with the kit. The cDNA was used as a template for semi-quantitative RT-PCR. The primers used are listed in Table 1.

**Table 1:** **List of primers used in the study**.

**Primer**	**Sequence**	**Restriction site**	**Tm (°C)**
alg8_fwd	TT**CTGCAG**AACTTACAAACGTGGCCTCG	*Pst*I	58–60
alg8_rev_RT	TT**GGATCC**CACGTGGAGGAACAGCATG	*BamH*I	58–60
16 s rRNA_fwd	AAGCAACGCGAAGAACCTTA	–	58–60
16 s rRNA_rev	CACCGGCAGTCTCCTTAGAG	–	58–60
pslA_fwd	TT**CTCGAG**ATCGAGTACTTCCTGGTCGC	*Xho*I	58–60
pslA_rev_RT	TT**AAGCTT**CGGTTGCTGAAGATATCGTCG	*Hind*III	58–60
alg8_up_fwd	TT**CTCGAG**GCCTGCTCGGCCTGTCGTT	*Xho*I	65–68
alg8_up_rev	TT**AAGCTT**CAGTTCCATCGGGCTGGGG	*Hind*III	65–68
alg8_dw_fwd	TT**CTGCAG**CCACCGAATCGACTACGGA	*Pst*I	60–62
alg8_dw_rev	TT**GGATCC**TTGGTGAAGTTCTCGCGCT	*BamH*I	60–62
KanSacI F	ATT**GAGCTC**TTAGAAAAACTCATCGAG	*Sac*I	55–62
KanSacI R	ATT**GAGCTC**ATGAGCCATATTCAACGG	*Sac*I	55–62

*Nucleotides in bold represent restriction sites*.

### Quantitative PCR

The cDNA prepared as mentioned above was subjected to quantitative PCR (qPCR) using SYBR GREEN reaction mix in a 7900HT Fast Real-Time PCR System (Applied Biosystems, USA). Thermal cycling conditions were as follows: 10 min at 95°C followed by 40 repeats of 30 s at 95°C, 30 s at 58°C. Following PCR amplification, the reactions were subjected to temperature ramping to create the dissociation curve, measured in terms of changes in fluorescence intensity as a function of temperature, by which non-specific products can be detected. The dissociation program was 95°C for 1 min, 60°C for 10 s, and 95°C for 30 s. The experiment included three biological replicates and each biological replicate was evaluated by three technical replicates. The constitutively expressed gene of *Pseudomonas* sp., for 16s rDNA, was taken as the calibrator in order to normalize gene expression levels. The MIQE guidelines (Bustin et al., 2009) for qPCR recommend the more generic quantification cycle (*C*_*q*_). However, as *C*_*t*_ and *C*_*q*_ are used interchangeably and refer to the same value, C_*t*_ was used in this study. The *C*_*t*_-values obtained in the experiment were used for quantification of relative change in gene expression, by the 2^−ΔΔCt^ method (Livak and Schmittgen, 2001).

### Alginate quantification

Alginate content in EPS was estimated as per the protocol described by Richardson et al. (2004). Briefly, to 1 mL of purified EPS, 1 mL of 0.8 M NaOH was added and neutralized with 120 μL 2.25 M citric acid after 5 min of incubation. To this, 40 μL of DMMB (1,9-dimethyl methylene blue) was added. The solution was vortexed vigorously and incubated at room temperature for 45 min. Thereafter, UV-visible spectrum of the samples was recorded between 500 and 700 nm using microtiter plate reader (Ultramark, Bioplate Imaging system, Biorad, USA). The absorbance intensities at 520 and 650 nm, signifying alginate-bound and unbound DMMB were used to estimate the respective alginate concentrations.

### Generation of *alg8* knockout mutant

The *alg8* gene knockout strategy involved gene replacement by homologous recombination, wherein the native *alg8* gene was replaced with an antibiotic resistance (*kan*^r^) cassette. For homologous recombination, a construct consisting of *kan*^r^ gene flanked by the regions upstream and downstream to *alg8* gene was generated, providing homology, as shown in Figure S2A. To assemble this construct, primers were designed to amplify ~500 bp upstream and downstream regions of *alg8* from strain Psd, as *Xho*I/*Hind*III and *Pst*I/*Bam*HI fragments, respectively. The primer sequences have been mentioned in Table 1. These fragments were sequentially cloned in pBlueScript (pBKS+) vector, using the aforementioned restriction sites to generate a construct termed as pBKS_*alg8*_up/down. Subsequently, *kan*^r^, was cloned between the upstream and downstream regions. The recombinant plasmid, pBKSKmΔAp (~3.3 kb) carrying kanamycin resistance gene, *kan*^r^ (~800 bp) cloned in *Sac*I restriction site, served as the source of antibiotic resistance gene. The vector construct, pBKS_*alg8*_up/down was digested with *Eco*RV to generate a blunt-ended linear DNA fragment. Both of these fragments were then ligated and transformed into *E. coli* XL1-Blue to generate a construct carrying *kan*^r^ gene flanked by regions upstream and downstream to *alg8* (pBKS_*alg8*_up/kan/down). Screening of transformants was done on LB Agar supplemented with 50 μg mL^−1^ of kanamycin. This was followed by plasmid isolation and PCR amplification of three regions, viz. upstream, downstream and *kan*^r^ using their respective primers. Amplification profile confirmed successful cloning of these regions in pBKS+ (Figure S2C). One of the positive constructs was transferred to strain Psd by electroporation and putative transformants were selected on kanamycin-containing agar medium. Due to the narrow host-range of pBKS+, the construct pBKS_*alg8*_up/kan/down was less likely to be maintained as a plasmid in strain Psd. Hence, growth of clones on kanamycin-supplemented medium is explained by the integration of *kan*^r^ cassette in place of *alg8* gene through homologous recombination (Figure S2B). In order to confirm stable insertion of *kan*^r^ cassette, positive transformants were transferred to non-selective conditions (without kanamycin) and grown for a few generations. Transformants with a stable integration were able to resist kanamycin when transferred back to the selection medium containing kanamycin.

### Assessment of plant-growth promoting and biocontrol parameters

Assessment of phosphate solubilization was carried out as per the protocol of Pikovskaya (1948). Extraction and detection of IAA was carried out using Ultra Performance Liquid Chromatography (UPLC), according to the modified protocol of Malhotra and Srivastava (2008). The secretion of siderophores was observed by spectral analysis at 400 nm (Schwyn and Neilands, 1987). Phenazine was extracted and quantified by the method described by Whistler and Pierson (2003). The antifungal activity of the strain was tested against two known plant pathogenic fungi, namely, *Fusarium oxysporum* and *Fusarium graminearum* by the following assays:

- Dual culture assay
  For dual culture assay, the Potato Dextrose Agar (PDA) plate was divided in two-halves. Inhibition assays were carried out by placing an agar-block from fully grown fungal plate on one half of the plate and streaking the biocontrol bacterium on the other half. The plates were incubated at 28°C for 5-days. Fungal inhibition was obtained as a zone of clearance on the plate in the area where bacteria were growing.
  The plates with bacterial growth and fungal block alone were taken as reference.
- Biomass inhibition
  For quantitative evaluation of antifungal activity, ~10^6^ spores of the fungi, *F. oxysporum* and *F. graminearum* were inoculated in PD medium diluted with bacterial culture extract in 1:1 ratio. The flasks were incubated at 28°C, 120 rpm for 4-days. Antifungal nature was demonstrated by comparing the percent dry weight of the treated fungal biomass in comparison to the untreated control. All untreated controls were grown in 50% diluted PD medium with standard succinate medium (SSM).

### Biofilm formation

Biofilm formation was measured using microtiter plate biofilm assay (Merritt et al., 2005). Briefly, 1% of the overnight grown cultures were subcultured in 10 mL GMM supplemented with increasing Zn^2+^ concentrations (0, 1, 2, 5 mM). From this, 100 μL culture was pipetted in a fresh 96-well microtiter plate. The plate was covered and incubated at 30°C for 48 h under static conditions. After incubation, the wells were washed thoroughly with distilled H_2_O to remove planktonic bacteria. Following this, 125 μL of crystal violet stain (0.1%) was added to each well. The stain was removed after 10 min of incubation at room temperature and plates were allowed to air-dry. Thereafter, 200 μL of 95% ethyl alcohol was added to each stained well in order to solubilize the dye. The contents of each well were mixed well and 125 μL of the crystal violet/ethyl alcohol solution was transferred to a fresh microtiter plate. The absorbance at 560 nm was measured using a microtiter plate reader (Ultramark, Bioplate Imaging system, Biorad, USA). All values were normalized with cellular OD_600_.

### Bacterial inoculation of seeds

Wheat (*Triticum aestivum* var. HD2851, IARI, New Delhi) seeds were surface sterilized with 0.1% (w/v) HgCl_2_ for 5 min and washed thoroughly with sterile distilled water. For bacterial inoculation, stationary phase bacterial cells, raised in GMM supplemented with different Zn^2+^ concentrations, were washed twice with saline to remove the residual medium and re-suspended in saline at a cell density of ~10^8^ cfu mL^−1^. To this suspension, 10 surface-sterilized wheat seeds were added and flasks were incubated at 30°C, 70 rpm for 2 h. After this treatment, seeds were thoroughly rinsed with sterile water. Seeds were sown in pots and growth of seedlings was monitored for 6-days after treatment.

### Confocal laser scanning microscopy

Confocal laser scanning microscopy of root samples from 6-day-old plantlets was performed according to the modified protocol by Bianciotto et al. (2001). Briefly, the root samples from 6-day-old plantlets were excised and washed with 100 mM cacodylate buffer (pH 7.40). Thereafter, the samples were stained with LIVE/DEAD® *Bac*Light™ Bacterial Viability Kit (ThermoFisher Scientific, USA) for 25 min at room temperature. The kit contains a mixture of SYTO 9 and propidium iodide stains, with excitation/emission maxima of 480/500 and 490/635 nm, respectively. After incubation at room temperature for 15 min, samples were mounted on clean glass slides using the mounting oil provided in the kit and observed under Leica TCS SP5 confocal microscope (Leica Microsystems, Germany).

### Scanning electron microscopy

Scanning electron microscopy (SEM) of root samples from 6-day-old plantlets was performed as described by Koul et al. (2015). Briefly, root samples (1–1.5 cm) were fixed with 2.5% gluteraldehyde in 0.1 M phosphate buffer for 6 h at 4°C. Following this, post-fixation was done with osmium tetraoxide (1% v/v in 0.1 M phosphate buffer) for 30 min. Thereafter, the specimens were washed thrice with milliQ water and treated with 2% (w/v) uranyl acetate for 40 min. The samples were dehydrated through a graded ethanol series (30–100%) followed by acetone (100%). The treated specimens were mounted on aluminum stubs, coated with gold-palladium and examined under a scanning electron microscope (EVO MA10, Carl Zeiss).

### Zn^2+^ estimation

Zn^2+^ content was estimated using atomic absorption spectrophotometer (Perkin Elemer model AAnalyst400) at 219.86 nm, as described earlier (Upadhyay and Srivastava, 2014). Flow diagram of the detailed protocol is shown in Figure S3.

### Statistical analysis

All bacterial culture experiments were carried out in three independent sets, each consisting of three replicates. Values shown here represent mean ± standard deviation (*SD*). All bacterial inoculation experiments were carried out in three independent sets containing 10 seeds each and values shown are mean ± *SD*. Data were tested at a significance level of *P* < 0.05 using one-way ANOVA followed by Dunnett's *t*-test and expressed as mean ± *SD*.

## Results

### Zn^2+^ accumulation induces ultrastructural changes

**Figure 1:**
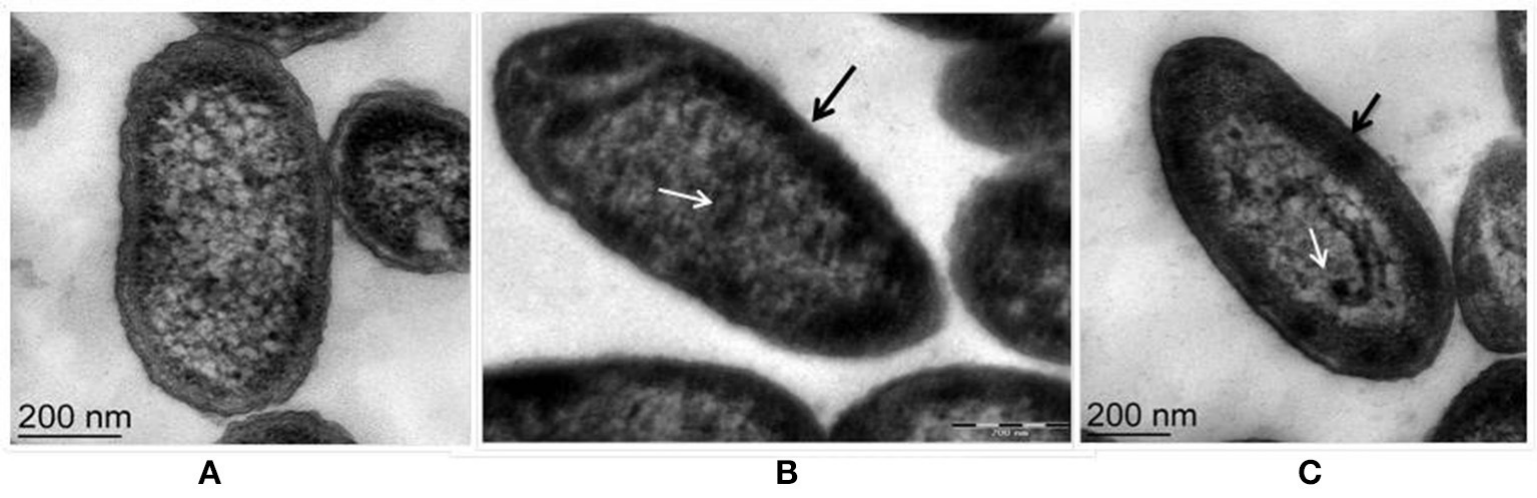
**Transmission Electron Microscopy images of ***Pseudomonas*** strain Psd exposed to varying Zn^**2+**^ concentrations in GMM for 24 h. (A)** Untreated control; Cells exposed to **(B)** 2 mM Zn^2+^ and **(C)** 5 mM Zn^2+^, respectively. Black arrows indicate thickening of cell wall due to metal biosorption. White arrows indicate intracellular accumulation by cytoplasmic granules (scale bar = 200 nm).

Fluorescent *Pseudomonas* strain Psd can sustain an external Zn^2+^ concentration of 5 mM (Upadhyay and Srivastava, 2014). We have used transmission electron microscopic (TEM) analysis to monitor the changes induced by Zn^2+^ accumulation by strain Psd. In comparison to cells growing without extra Zn^2+^ supplementation in the medium (Figure 1A), cells exposed to increasing Zn^2+^ concentration displayed thickening of the outer membrane, which pointed toward extracellular biosorption of Zn^2+^ on to the cell surface (Figures 1B,C). In addition to the extracellular accumulation, some electron dense aggregates (EDA) were also found in the cytoplasm of the cells. Although most of the Zn^2+^ is located on the outer surface, internally cytoplasmic granules may provide additional sites for deposition of the heavy metal.

### FT-IR spectrum of the EPS revealed the presence of characteristic functional groups

**Figure 2:**
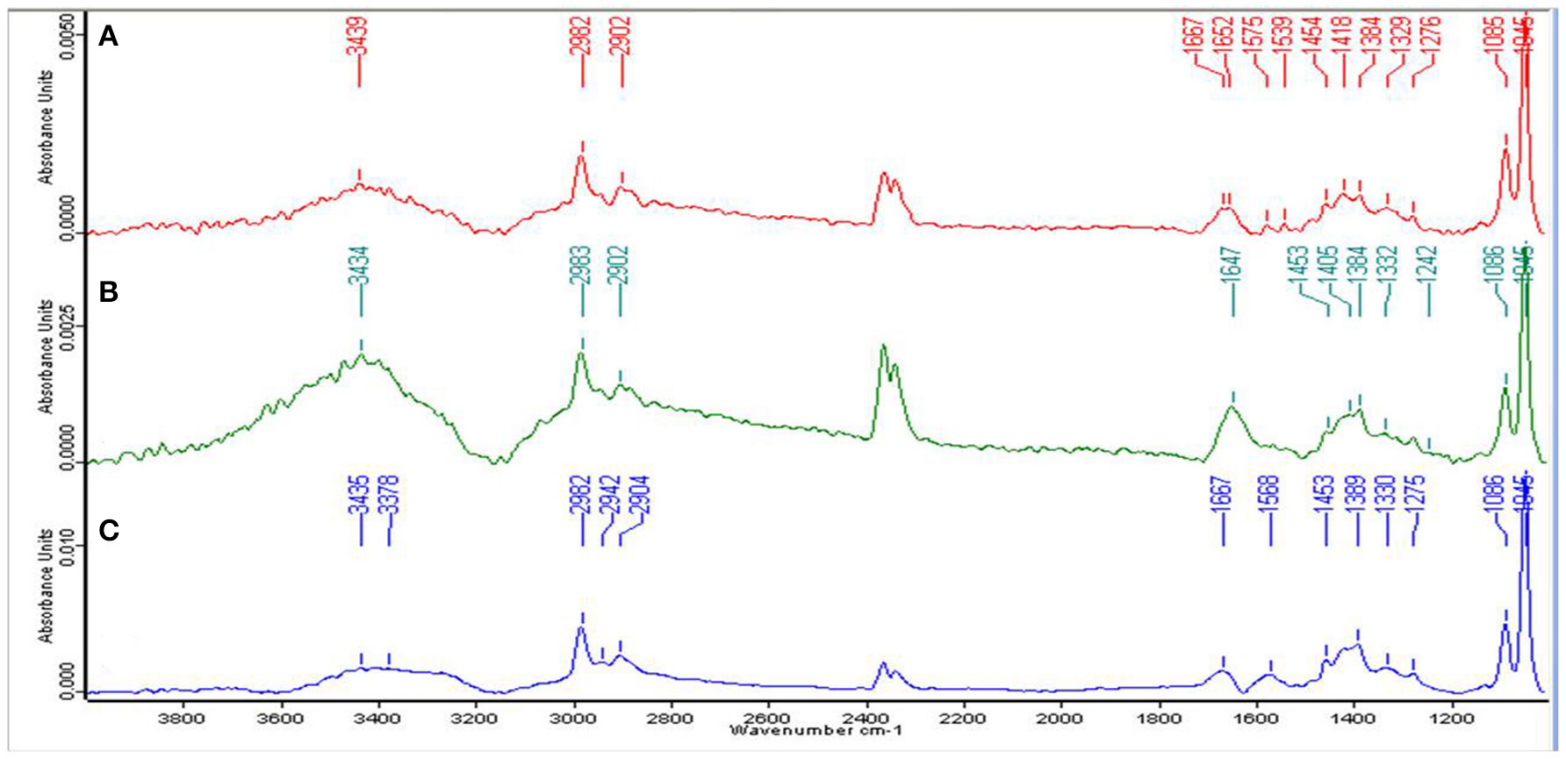
**Fourier-transformed infrared (FTIR) spectrum of the exopolysaccharide produced by Psd grown in the presence of varying Zn^**2+**^ concentrations in Gluconate Minimal Medium**. **(A)** Untreated control; Cells grown in the presence of **(B)** 2 mM Zn^2+^, and **(C)** 5 mM Zn^2+^, respectively.

For the qualitative analysis of functional groups facilitating Zn^2+^ biosorption, EPS from the cells of strain Psd grown at increasing Zn^2+^ concentrations was subjected to FT-IR spectroscopy. The presence of functional groups such as C-O and C-O-C, signified by stretching between 1,200 and 1,000 cm^−1^ and O-H, signified by elongation between 3,700 and 3,200 cm^−1^, was indicative of the presence of carbohydrates, which is the major component of the EPS biopolymer. Sharp peaks in the range of 1,125 to 1,000 cm^−1^ indicated toward the presence of uronic acids in the EPS. The spectrum also revealed the presence of mannose in the EPS, signified by a peak at ~2,900 cm^−1^. The mannose peak, however, was weaker in comparison to the uronic acids. Besides, the peaks in the range of 1,210 to 1,140 cm^−1^ revealed the presence of phosphoryl groups in the EPS. The presence of protein in the EPS was also implicated. Generally, proteins are detected by C = O (Amide I) stretching between 1,680 and 1,630 cm^−1^, N-H bending vibration (Amide II) between 1,650 and 1,550 cm^−1^ and N–H stretching (Amide A) vibration between 3,290 and 3,300. Peaks corresponding to Amide I and Amide II bonds were observed in untreated control and cells exposed to 2 mM Zn^2+^ (Figures 2A,B). In the cells exposed to 5 mM Zn^2+^, however, only peak corresponding to Amide I bond was observed (Figure 2C).

### Zn^2+^ replete conditions affect *alg8* expression

**Figure 3:**
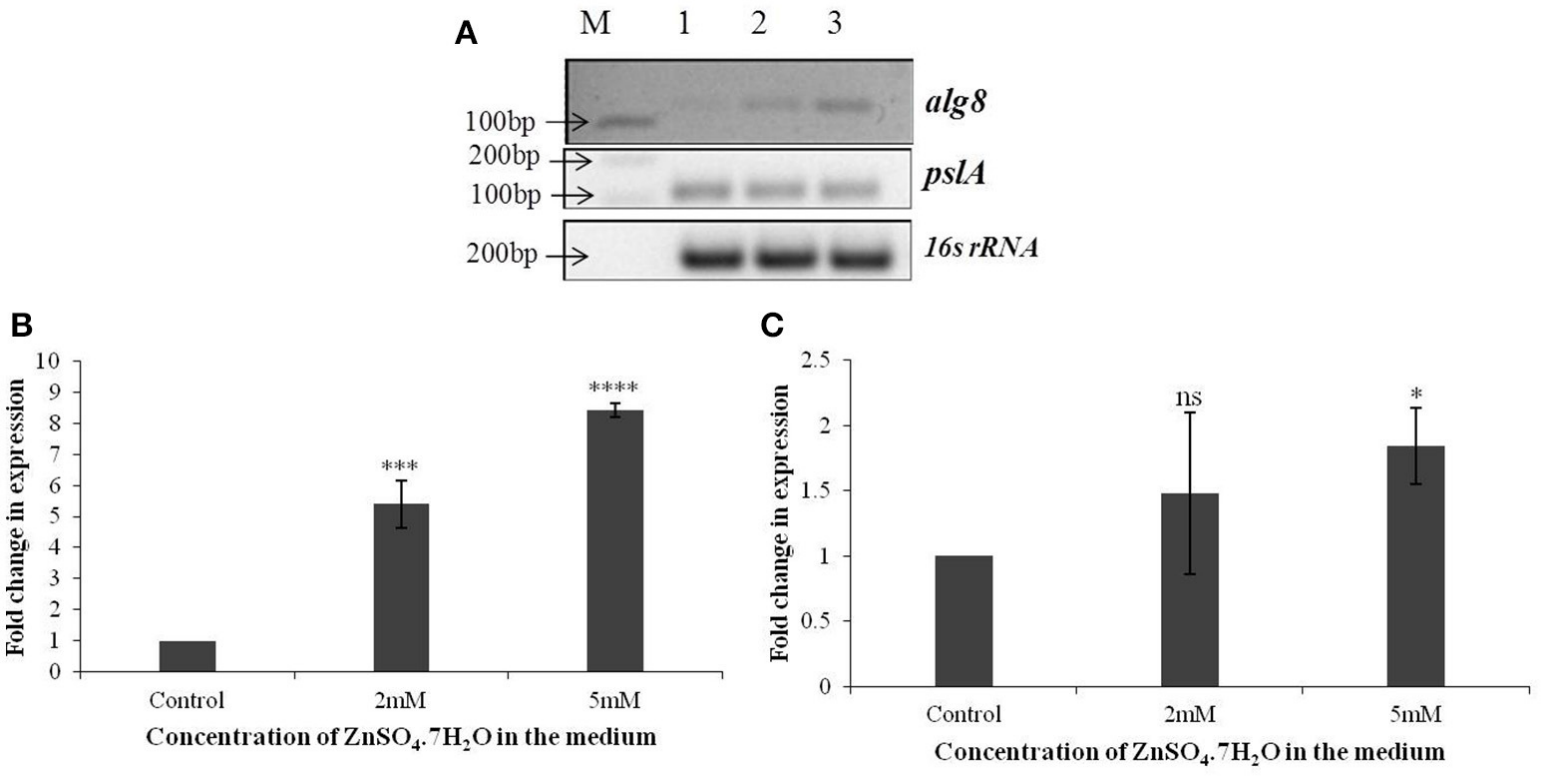
**(A)** Semi-quantitative-RT PCR of *alg8* and *pslA* involved in exopolysaccharide biosynthesis in *Pseudomonas* strain Psd exposed to varying Zn^2+^ concentrations (M-1 kb DNA ladder, Lane 1 represents strain Psd grown in the absence of added Zn^2+^; Lanes 2 and 3 represent strain Psd grown at 2 and 5 mM Zn^2+^, respectively); **(B)** Fold-change in the expression of *alg8* in strain Psd in response to exposure to increasing Zn^2+^ concentrations; **(C)** Fold-change in the expression of *pslA* in strain Psd in response to exposure to increasing Zn^2+^ concentrations. ^*^P < 0.05; ^***^P < 0.001; ^****^P < 0.0001; ns, not significant.

Based on the identification of uronic acids and mannose by FT-IR spectroscopy, the relative expression of two genes, *alg8* and *pslA*, involved in biosynthesis of two principle exopolysaccharides, alginate and Psl, were studied in strain Psd exposed to varying Zn^2+^ concentrations, by real-time PCR using the gene encoding 16s rRNA as the reference gene or calibrator. The *alg8* gene codes for alginate polymerase and *pslA*, encoding a sugar transferase, is responsible for the production of the mannose and galactose-rich Psl polysaccharide. When compared to the untreated control, a significant up-regulation was obtained in *alg8* expression in the presence of Zn^2+^ (Figures 3A,B). Cells exposed to 5 mM Zn^2+^ displayed 8-fold increase in *alg8* expression when compared to control or untreated cells (Figure 3B). On the other hand, no significant changes in the expression of *pslA* were obtained between control and Zn^2+^—grown cells (Figures 3A,C).

### Zn^2+^ accumulation is coupled with higher alginate production

Based on the above results, it was concluded that alginate is the principle component of EPS produced by strain Psd as a direct correlation with its exposure to varying concentrations of Zn^2+^, and, thus may be involved in Zn^2+^ biosorption. To confirm this, spectrophotometric quantification of alginates, based on 1,9-dimethyl methylene blue (DMMB) complexation was used to estimate the amount of alginates in the EPS produced by the bacterial cells growing in different Zn^2+^ concentrations. As shown in Figure S4, strain Psd was able to produce 0.6 mg/mL of alginates after 5-days of incubation. In the presence of Zn^2+^, up to 3-fold increase was recorded in the amount of alginate produced. This result complied with the earlier hypothesis of the involvement of alginate in Zn^2+^ biosorption by strain Psd. Further evidence for the same was provided by generation of an *alg8* knockout of strain Psd.

### Deletion of *alg8* reduces Zn^2+^ biosorption potential

In order to ascertain the involvement of *alg8* during Zn^2+^ biosorption by strain Psd, it was important to generate a mutant strain devoid of this function. The *alg8* gene knockout strategy involved gene replacement by homologous recombination, wherein the native *alg8* gene was replaced with an antibiotic resistance (*kan*^r^) cassette (Figure S2).

**Figure 4:**
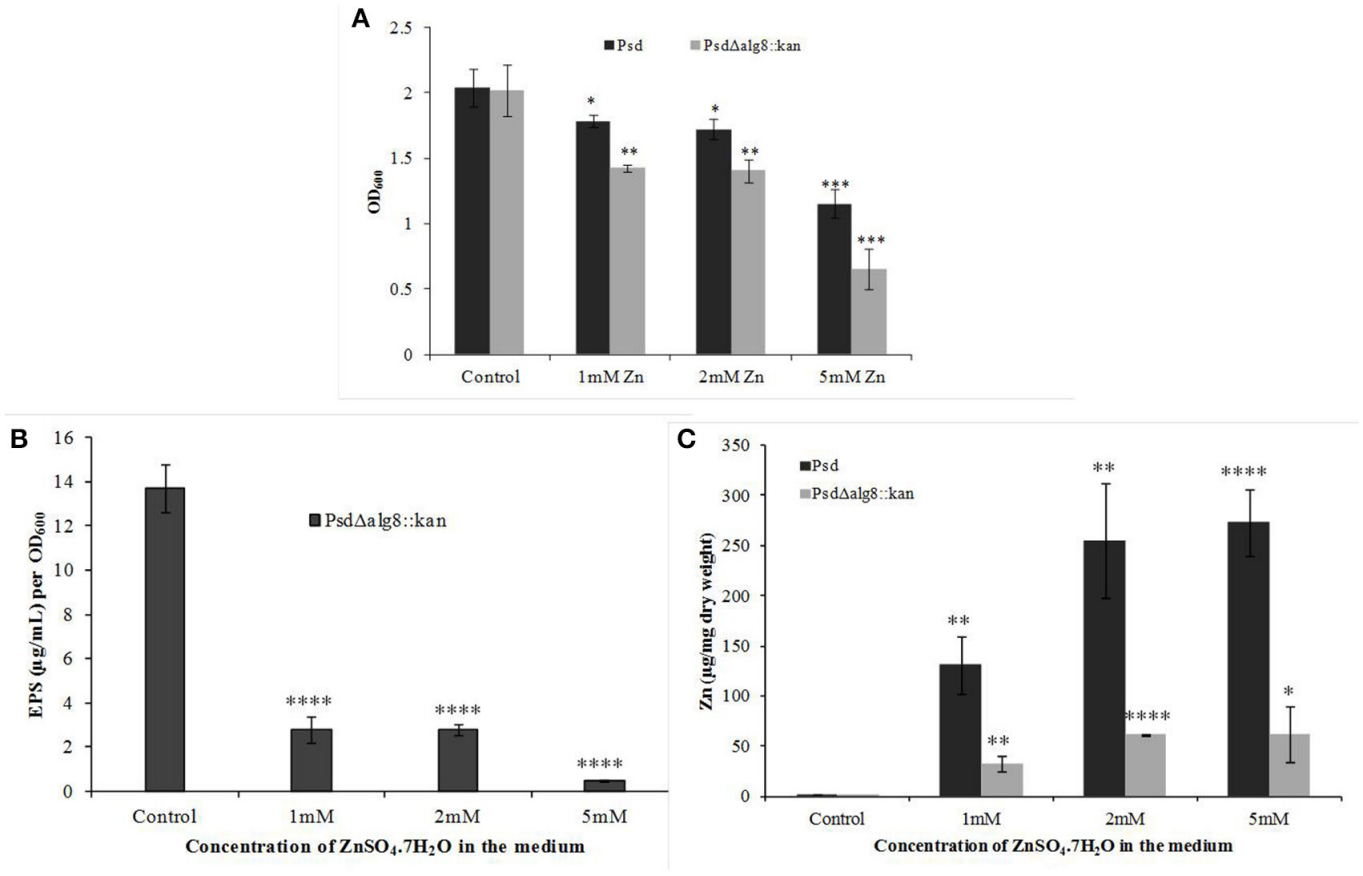
**Effect of deletion of ***alg8*** on (A)** Net growth **(B)** EPS production and **(C)** Zn^2+^ accumulation by strain Psd (^*^p < 0.05, ^**^p < 0.01, ^***^p < 0.001, ^****^p < 0.0001).

The mutant strain Psd Δ*alg8*::*kan* had a similar growth profile as the wild-type strain Psd in the absence of Zn^2+^. This showed that growth of the mutant strain was not affected by insertion of the kanamycin cassette. Supplementation of medium with 2 mM Zn^2+^, however, led to up to 20% drop in cell viability of the mutant. The survival further declined with the increase in Zn^2+^ concentration to 5 mM, wherein, 44% decrease in cell survival was obtained (Figure 4A). Besides, *alg8* deletion resulted in 80% decrease in the levels of EPS synthesized at external Zn^2+^ concentrations of 2 mM, which increased to 97% at 5 mM Zn^2+^ (Figure 4B). Further, since the EPS production in the *alg8*-negative mutant was compromised, Zn^2+^ accumulation by the mutant was compared with that of the wild-type strain. For this purpose, the mutant strain (Psd Δ*alg8*::*kan*) was grown in GMM supplemented with different Zn^2+^ concentrations (1, 2, 5 mM) for 24 h, along with a set containing no Zn^2+^. Wild-type strain Psd was used as control. It was observed that besides a reduced EPS secretion, the deletion of *alg8* also led to a drastic decrease in Zn^2+^ accumulation potential of the strain (Figure 4C).

**Figure 5:**
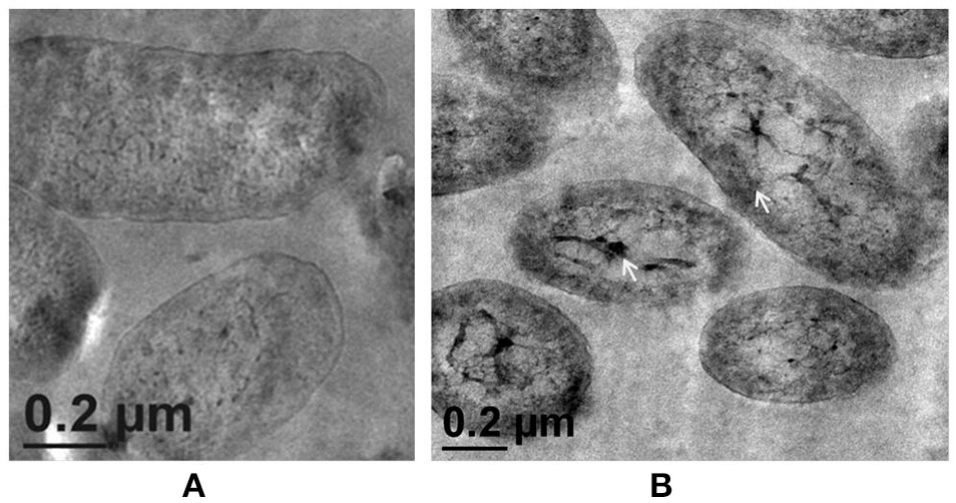
**Transmission Electron Microscopy images of strain PsdΔ***alg8***::***kan*** exposed to varying Zn^**2+**^ concentrations in Gluconate Minimal Medium. (A)** Untreated control; **(B)** Cells exposed 5 mM Zn^2+^.The samples were taken after 24 h of growth in Gluconate Minimal Medium. White arrows indicate the intracellular Zn^2+^ accumulation in the form of cytoplasmic granules (scale bar = 200 nm).

Morphological examination of the mutant cells through TEM revealed a comparatively thin outer membrane along with alterations in membrane architecture, supporting the above observations. There was an evident reduction in the cell wall thickening in the untreated control cells as compared to the wild-type strain (Figure 5A, also refer Figure 1). Further, exposure of cells to higher Zn^2+^ concentrations did not show traces of extracellular accumulation of Zn^2+^ (Figure 5B). However, cytoplasmic granules were observed in the presence of extracellular Zn^2+^, indicating that intracellular Zn^2+^ entry was not affected (Figure 5B). This was suggestive of a regulated intracellular entry of Zn^2+^ in the strain. When EPS is present, the cell is able to keep the high concentration of Zn^2+^ outside. Taken together, all these observations supported the fact that *alg8* plays an important role in EPS production by strain Psd and is, also, the primary component involved in Zn^2+^ biosorption by the strain.

### Biofilm formation and *in vitro* root colonization

Formation of static biofilms was analyzed by crystal violet binding assay. As shown in Figure S5, increased Zn^2+^ concentration aided in biofilm formation by the strain. This was in accordance with the earlier observation that Zn^2+^ biosorption led to increased EPS biosynthesis by the strain. On the other hand, PsdΔ*alg8*::*kan* produced significantly low amount of biofilm as compared to wild-type. This could be attributed to compromised EPS production by the mutant.

**Figure 6:**
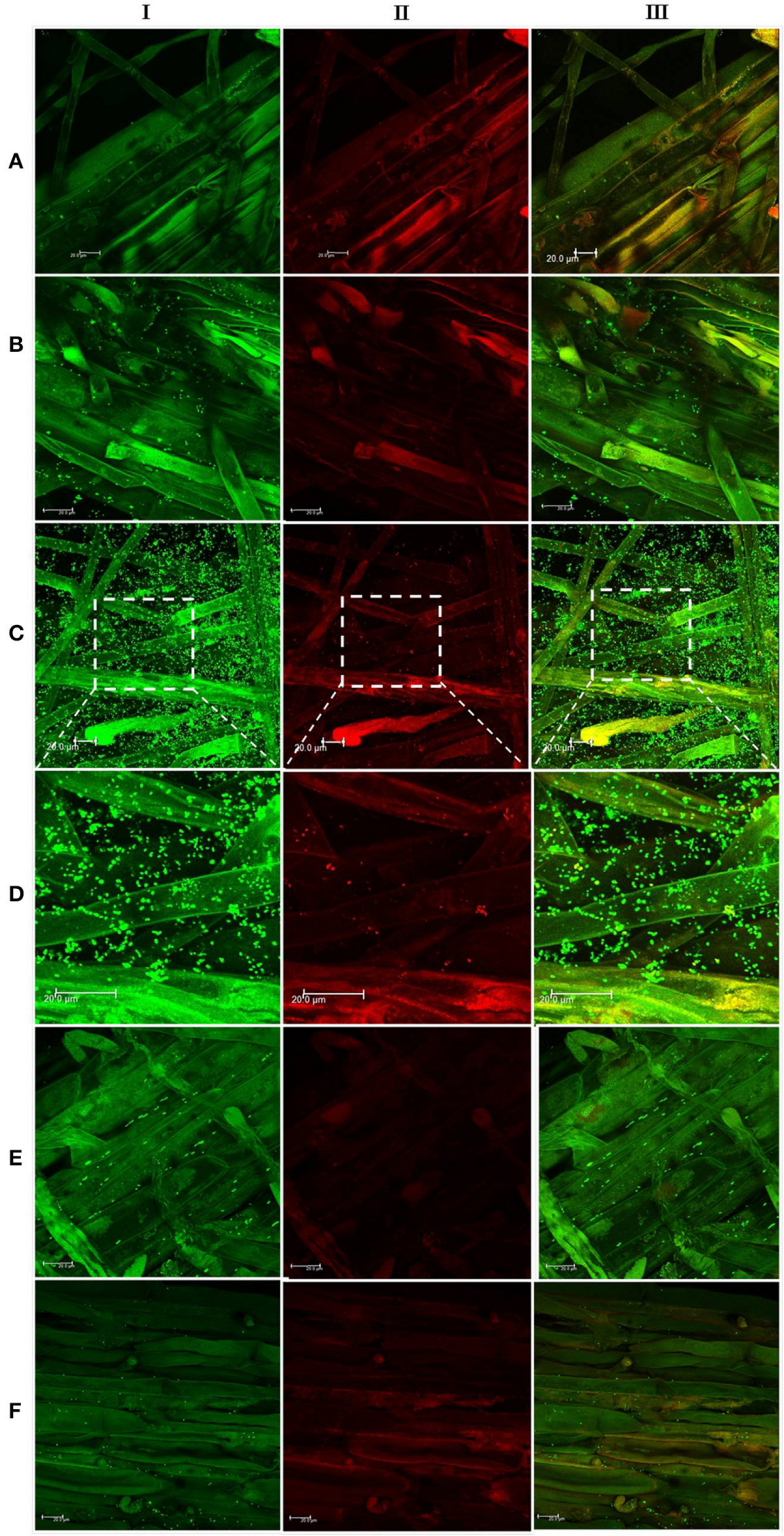
**Confocal laser scanning microscopy images of roots from 6-day-old plantlets of ***Triticum aestivum*** in the presence of strain Psd and its ***alg8*** deletion mutant**. Untreated control **(A)**; roots treated with wild type strain Psd grown in the absence of Zn^2+^ **(B)**; roots treated with wild type strain Psd grown in the presence of 2 mM Zn^2+^ **(C)**; enlarged section of panel C **(D)**; roots treated with PsdΔ*alg8*::*kan* grown in the absence of Zn^2+^; **(E)** roots treated with PsdΔ*alg8*::*kan* grown in the presence of 2 mM Zn^2+^ **(F)**. The columns I, II, and III represent Syto 9, propidium iodide stained and merged versions of samples. Bacteria are visible as small green dots on the root surface. Scale bar = 20 μm.

**Figure 7:**
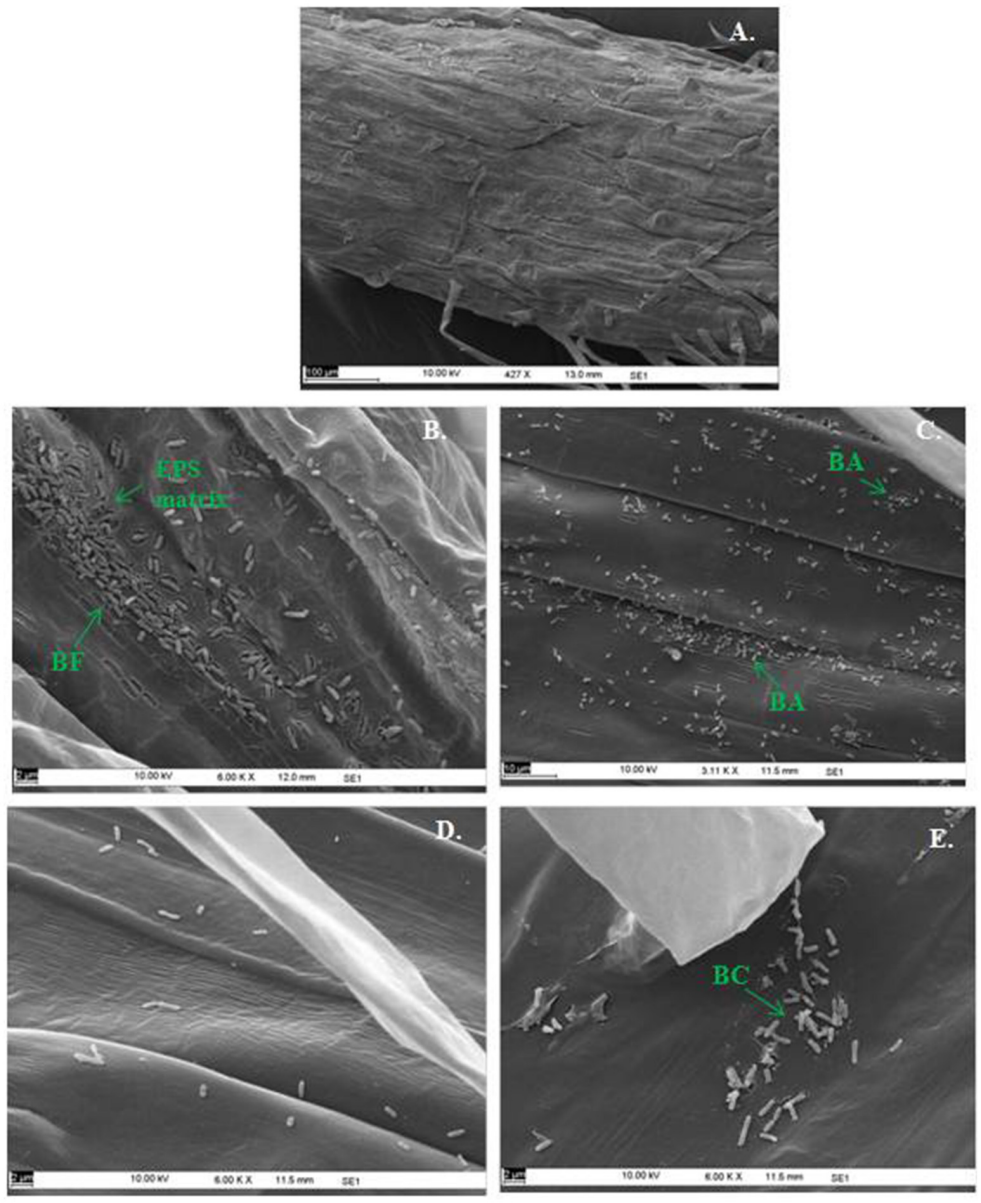
**Scanning Electron Microscopy of ***Triticum aestivum*** roots inoculated with strain Psd and its ***alg8*** deletion mutant to show the effect on roots:** Untreated control **(A)**, roots treated with wild type strain Psd grown in the absence of Zn^2+^; **(B)** roots treated with wild type strain Psd grown in the presence of 2 mM Zn^2+^ **(C)**, roots treated with PsdΔ*alg8*::*kan* grown in the absence of Zn^2+^; **(D)** roots treated with PsdΔ*alg8*::*kan* grown in the presence of 2 mM Zn^2+^ **(E)**. BF, Biofilm; BC, Bacterial Clusters; BA, Bacterial aggregates. Scale bars vary from 2 to 100 μm and are shown in each image.

In the context of PGP bacteria, biofilm formation aids in effective root colonization, thus playing a crucial role in plant-microbe interactions. The association of strain Psd and its *alg8* deletion variant with 6-day-old plantlets of *T. aestivum* was visualized by means of confocal microscopy, followed by SEM. As shown in Figures 6A, 7A, no bacteria were observed on the control roots. On the other hand, the wild-type strain Psd was able to associate with the roots (Figure 6B), and form thick biofilms associated with EPS matrix (Figure 7B). Additionally, Zn^2+^ accumulation also aided formation of bacterial aggregates and biofilm (Figures 6C,D, 7C). In case of the mutant strain, however, biofilm formation was drastically reduced, and mostly planktonic cells were observed on the roots (Figures 6E,F, 7D,E). In certain cases, however, presence of bacterial clusters was detected (Figure 7E).

### Effect of *alg8* deletion on PGP potential, biocontrol activity, and antifungal potential

**Figure 8:**
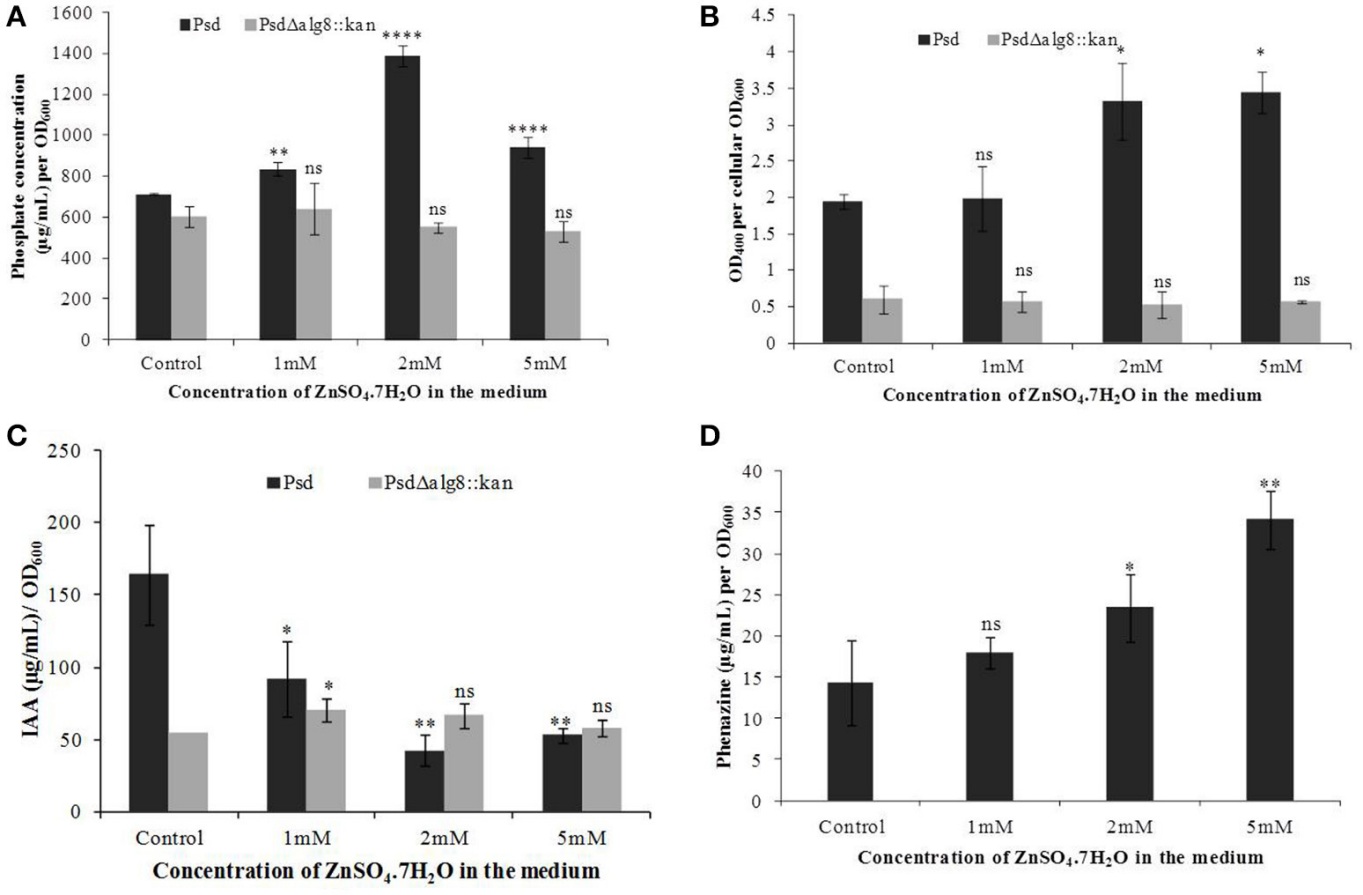
**Quantification of (A)** Phosphate solubilization **(B)** Siderophore production, **(C)** IAA production, and **(D)** Phenazine biosynthesis by Psd and PsdΔ*alg8*::*kan* in GMM supplemented with increasing Zn^2+^ concentrations. ^*^P < 0.05, ^***^P < 0.001, ^****^P < 0.0001, ns, not significant.

In continuation with the observation that *alg8* deletion severely affects the biofilm formation by the strain Psd, we were interested in observing its subsequent effect on the PGP potential. Increased accumulation of Zn^2+^ by strain Psd interestingly led to an increase in the phosphate-solubilization and siderophore production by the strain (Figures 8A,B). On the other hand, in strain Psd Δ*alg8*::*kan*, both these activities were severely compromised. A different response was observed during the assessment of IAA production. In the absence of added Zn^2+^ in the medium, a 24 h-grown culture of strain Psd produced 164 μg IAA/OD_600_. With increase in medium Zn^2+^ concentration, however, there was a significant drop in the amount of IAA produced. Strain Psd grown in the presence of 2 and 5 mM Zn^2+^ concentrations produced ~70% less IAA than the control. The mutant PsdΔ*alg8*::*kan* produced ~67% less IAA than the wild-type strain Psd in the absence of Zn^2+^ (Figure 8C). Zn^2+^ supplementation, however, did not have a significant effect on IAA produced by the mutant. Increased Zn^2+^ accumulation also had a stimulatory effect on phenazine production by strain Psd (Figure 8D). This indicated that Zn^2+^ accumulation by strain Psd will enhance its biocontrol activity. On the other hand, in PsdΔ*alg8*::*kan*, no phenazine production was detected.

**Figure 9:**
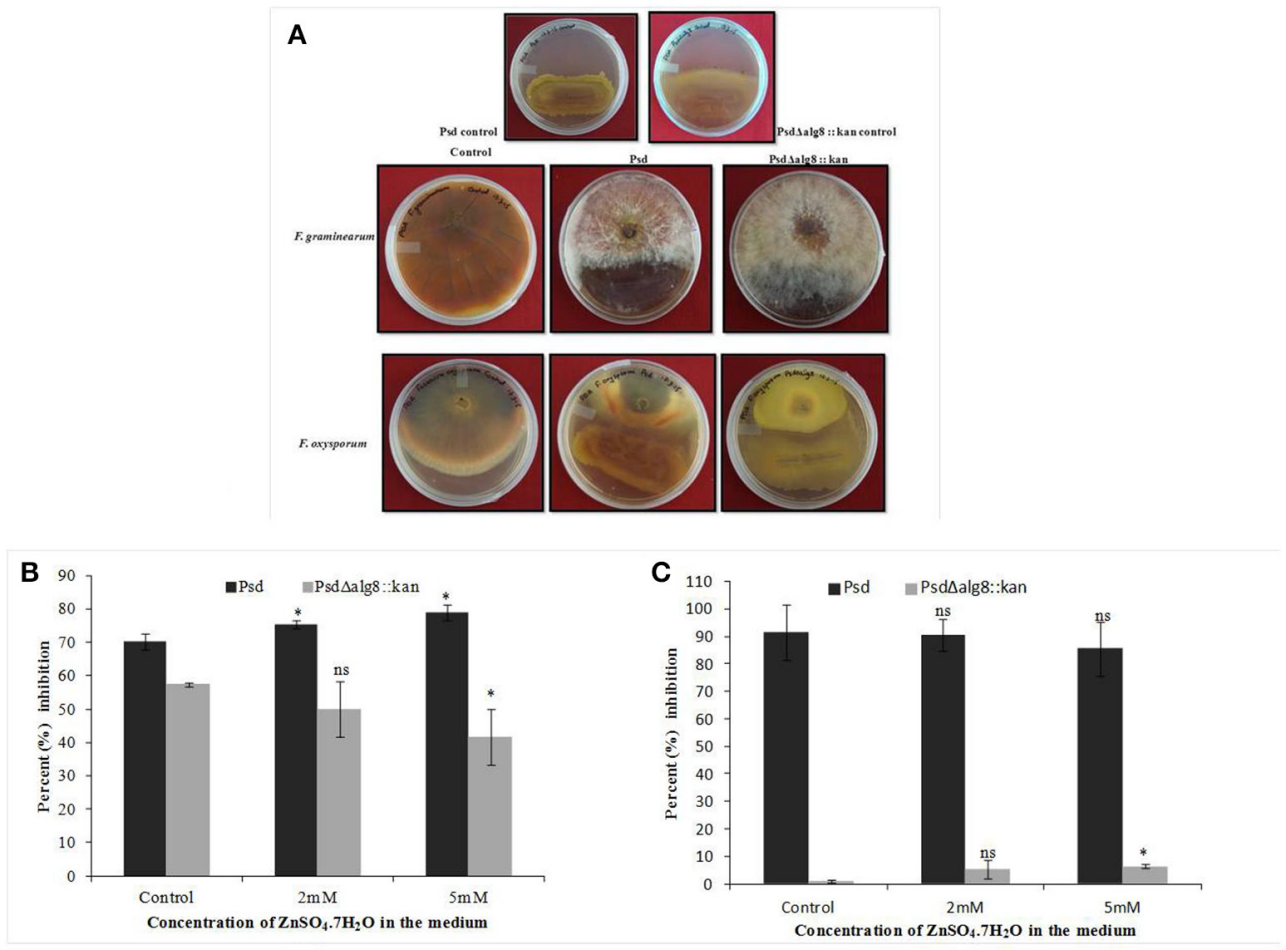
**(A)** Dual culture assay to show inhibition of *Fusarium graminearum* and *F. oxysporum* by strains Psd and PsdΔ*alg8*::*kan*. Effect of culture filtrate of strains Psd and PsdΔ*alg8*::*kan* on the biomass growth of **(B)** *F. oxysporum*, and **(C)** *F. graminearum* (^*^P < 0.05, ns-not significant).

The secondary metabolites produced by *Pseudomonas* sp. contribute to their biocontrol properties by inhibition of phytopathogens. Strain Psd showed an increase in the production of siderophores and phenazine in the presence of added Zn^2+^ in the medium, suggesting that Zn^2+^ modulates the production of these crucial secondary metabolites. On the contrary, deletion of *alg8* resulted in decreased production of Fe^3+^-chelating siderophores by the strain. Additionally, phenazine production was also hampered in the *alg8* deletion mutant. The major implication of these differences was reflected in the antifungal properties of strain Psd and its *alg8*-negative variant. Antifungal assays to test the ability of the strains to inhibit two phytopathogenic fungi, *F. oxysporum, F. graminearum*, comprised dual-culture tests followed by biomass inhibition experiments. Strain Psd was able to inhibit the growth of both the fungal strains growing on PDA (Figure 9A). In contrast, PsdΔ*alg8*::*kan* showed inhibition of only *F. oxyporum* to some extent. No inhibition, however, was obtained in the case of *F. graminearum*. This clearly indicated that both strains differed in their antifungal spectrum, and can be explained by the fact that these Pseudomonads are known to produce a variety of antifungal metabolites. To corroborate the above observation, inhibition of fungal biomass was studied. The culture filtrate from wild-type strain Psd led to 70 and 90% reduction in biomass of *F. oxysporum* and *F. graminearum*, respectively. The potential of strain Psd as an antifungal agent was supported by the observation that the culture filtrate was able to cause 70 and 90% reduction in biomass of *F. oxysporum* and *F. graminearum*, respectively (Figures 9B,C). Inhibition by strain Psd grown in the presence of added Zn^2+^ in the medium increased up to 80% in case of *F. oxysporum* (Figure 9B). No significant difference, however, was obtained in case of *F. graminearum* (Figure 9C). In contrast, inhibition by PsdΔ*alg8*::*kan* reduced to 60% in *F. oxysporum* (Figure 9B). The inhibition efficiency declined further with increase in Zn^2+^ concentration of the medium, with 40% inhibition at 5 mM Zn^2+^. The mutant strain was not able to inhibit *F. graminearum* at all (Figure 9C).

### Effect of *alg8* deletion on seedlings growth

The above results highlighted the Zn^2+^ accumulation potential of strain Psd and, subsequently, its beneficial effect on PGP and biocontrol potential of the strain. Furthermore, it was observed that this effect translated into improved plant growth. The treatment of wheat seeds with strain Psd improved root growth and proliferation, as is evident from Figure S6A. Zn^2+^-laden biomass of strain Psd led to improved seedlings growth as higher number of root hairs was observed in these cases (Figure S7). On the other hand, PsdΔ*alg8*::*kan*-treated wheat seeds did not show any differences with respect to the untreated control (Figure S6B), indicating that lack of alginate has affected the PGP potential of the strain.

## Discussion

Many members of fluorescent *Pseudomonas* sp., including *P. fluorescens* are inhabitants of the rhizosphere and possess the ability to enhance plant growth, via mechanisms such as phytohormone production (Upadhyay and Srivastava, 2010; Kochar et al., 2011; Ahemad and Kibret, 2014), biocontrol of phytopathogens by synthesizing allelopathic factors such as toxins, antibiotics, and siderophores (Duffy and Défago, 1999; Upadhyay and Srivastava, 2008, 2010; Couillerot et al., 2009), and improvement of nutrient acquisition by the plants (Upadhyay and Srivastava, 2008; Sirohi et al., 2015; Wang et al., 2015). Besides, these bacteria have also emerged as promising candidates for *in situ* bioremediation of various organic and inorganic pollutants, including heavy metals (Zhuang et al., 2007; Upadhyay and Srivastava, 2014). Fluorescent *Pseudomonas* sp. strain Psd, isolated from the rhizosphere of *V. mungo*, has been extensively studied and has been demonstrated to possess multiple plant-growth promoting and biocontrol potential (Upadhyay and Srivastava, 2008, 2010; Kochar et al., 2011). Besides, we have reported earlier that the Zn^2+^ accumulation by strain Psd is accompanied by increased EPS production (Upadhyay and Srivastava, 2014). We decipher here the role EPS plays in the process of biosorption of Zn^2+^ and also their implications on the PGP and biocontrol potential of the strain.

Exposure to metals induces morphological and ultrastructural changes in the cell. Membrane thickening and protuberances were observed in the cells of strain Psd exposed to high Zn^2+^ concentrations. These changes are likely to provide increased number of binding sites to accommodate high levels of Zn^2+^. In such cells, EDA which mediate intracellular accumulation of the metal were also observed. Similar results were obtained in *Pseudomonas stutzeri* RS34 exposed to Zn^2+^ (Bhagat and Srivastava, 1994). Changes in cell morphology in response to exposure to heavy metals has been reported in case of extremophiles *Acidocella, Acidiphillium*, and cyanobacterium, *Microcoleus chthonoplastes* that show greater excretion of EPS in response to exposure to heavy metals like Cd^2+^, Cu^2+^, Ni^2+^, Pb^2+^, and Zn^2+^ (Chakravarty et al., 2007; Diestra et al., 2007; Chakravarty and Banerjee, 2008). Further, profiling of functional groups present in the EPS secreted in response to exposure to Zn^2+^ revealed the presence of carbohydrates, uronic acids, mannose, and phosphoryl groups. Out of these components, uronic acids have been established as the main constituents of bacterial biofilms (Sutherland, 2001). Additionally, uronic acids were also found to form the major components of the EPS involved in the sequestration of cations such as Pb^2+^, Cd^2+^, Co^2+^, Ni^2+^, and Zn^2+^ and Cu^2+^ in *Paenibacillus jamilae* (Pérez et al., 2008). Several functional groups present on bacterial cell wall, including carboxyl, phosphonate, amine, and hydroxyl groups, which assist in biosorption have been identified (Ledin, 2000; Vijayaraghavan and Yun, 2008). Ueshima et al. (2008) have demonstrated the presence of carboxyl and phosphoryl groups in the EPS produced by *P. putida*, which leads to the biosorption of Cd^2+^. Glucose and mannose pre-dominated the EPS involved in Zn^2+^ and Cd^2+^ biosorption by *Anoxybacillus* sp. and Pb^2+^ and Hg^2+^ biosorption in *Azotobacter chroococcum* (Rasulov et al., 2013; Zhao et al., 2014).

Alginate forms the major component of the biofilms secreted by bacteria (Ghafoor et al., 2011). We found an increase in the total alginate content of the EPS isolated from cells growing at different Zn^2+^ concentrations, indicative of the role this polysaccharide may play in the process. Several multivalent cations, like Ca^2+^, Cd^2+^, Pb^2+^, and Zn^2+^, bind to EPS by virtue of electrostatic interactions, thus triggering extensive production of these polysaccharides (Sutherland, 2001). In order to further characterize the role of alginates, we studied a gene from the alginate biosynthesis complex, *alg8*, which codes for β-glycosyl transferases of the GT-1 family and is a key protein involved in alginate polymerization (Rehm, 2010; Ghafoor et al., 2011). Increased Zn^2+^ accumulation, accompanied by upregulation of *alg8* expression, confirmed that alginate, besides being responsible for biofilm formation, also has a role in Zn^2+^ biosorption by Psd. Although many recent studies have demonstrated the role of alginates in metal biosorption in algal species (Plazinski, 2013; Bertagnolli et al., 2014), only a few reports are available in the bacterial system for the same (Zhang et al., 2010; François et al., 2012). Further insight into the role of *alg8* in Zn^2+^ biosorption was obtained with the help of a mutant devoid of a functional *alg8* gene. Gene replacement via homologous recombination has been employed in the present study to obtain *alg8* knockout mutant in strain Psd. Similar methods have been used in other studies to generate *alg8* negative mutant (Remminghorst and Rehm, 2006; Ghafoor et al., 2011). These studies have shown that deletion of *alg8* results in non-mucoid phenotype, which is deficient in alginate production. None of the studies so far, however, have related deletion of *alg8* with metal tolerance. We have shown that the *alg8*-negative mutant had a compromised EPS production in comparison to the wild-type, ultimately affecting the Zn^2+^ accumulation potential of the strain. The mutant strain accumulated 80% less Zn^2+^ than the wild-type. This observation was further substantiated with the ultra-structural analysis, wherein the Zn^2+^- exposed cells of the mutant exhibited a clear reduction in the extracellular biosorption when compared to wild-type. Cytoplasmic granules, however, were observed in the mutant, indicating that the intracellular accumulation, if any, was not affected.

Biofilm formation by PGP bacteria is an important determinant in plant-microbe association. Formation of an extracellular matrix composed of EPS is the hallmark for biofilm formation (Wozniak et al., 2003). In the present study, we found a positive correlation between Zn^2+^ biosorption by strain Psd and alginate production. Hence, it was likely that inability of the strain to produce alginates would affect the strain's biofilm formation potential. It was, indeed, found that Zn^2+^ triggers the formation of biofilms by the strain. Microscopic and ultra-structural analysis revealed effective colonization of wheat roots by strain Psd, where the cells were found embedded in an extracellular matrix. Inability of the mutant PsdΔ*alg8*::kan to synthesize EPS affected its biofilm formation and as a consequence, cells were mostly found to exist as planktonic cells. However, we did find few aggregates on the mutant treated roots. This may be attributed to the fact that mutants unable to synthesize EPS may still attach to the surface and form micro-colonies to a limited extent (Sutherland, 2001).

Since strain Psd is a PGPB, it was essential to ascertain the effect of Zn^2+^ biosorption on its PGP potential. The present study has shown that the cells of strain Psd growing at high concentration of Zn^2+^ led to enhanced phosphate solubilization, which could be attributed to either enhanced EPS production at higher Zn^2+^ concentration which holds free P from insoluble phosphate in the medium (Ahemad and Kibret, 2014) or due to involvement of Zn^2+^ in conversion of glucose to gluconic acid (Ramachandran et al., 2006). The former is the most plausible explanation for decreased P solubilization activity of the *alg8* deletion mutant. Further, analysis of siderophores, which provide a competitive advantage to the biocontrol agents over harmful phytopathogens by limiting the supply of essential trace elements, revealed ~1.8-fold increase in siderophore production in Zn^2+^-grown cells of strain Psd. Exogenous environmental signals, like carbon sources and minerals, play an important role in modulating secondary metabolite production by microbes. It has been proposed that Zn^2+^ may hinder cellular iron uptake, leading to higher siderophore production. In *P. aeruginosa*, Zn^2+^ supplementation resulted in an overall iron deficiency (Rossbach et al., 2000). Alternatively, Zn^2+^ may bind to the siderophores, necessitating their enhanced production to chelate the available iron (Höfte et al., 1993). In agreement, significantly low levels of siderophores were detected in the mutant PsdΔ*alg8*::kan, due its compromised Zn^2+^ accumulation ability.

On the contrary to the above observations, in the present study, a decrease in IAA production was obtained with increased Zn^2+^ accumulation by strain Psd. This may be the consequence of lower IAA biosynthesis induced by Zn^2+^ or to auxin degradation by IAA peroxidases, which are up-regulated by metal-catalyzed free radical formation (Dimkpa et al., 2008). Besides, we found an increase in phenazine production with increased Zn^2+^ accumulation by strain Psd. This was in agreement with the observation of Duffy and Défago (1999), who reported the stimulatory effect of zinc sulfate on PHL and PLT production by *P. protegens* CHA0. Supplementation with Zn^2+^ also led to stimulation of phenazine-1-carboxylic acid production in *P. fluorescens* 2–79, which further improved the biocontrol potential of the strain (Slininger and Jackson, 1992). The exact mechanism for such a response is uncertain. However, it has been proposed that Zn^2+^ and other mineral nutrients stabilize the regulatory genes critical for antibiotic production in Pseudomonads (Duffy and Défago, 1997). Interestingly the alginate knock-out mutant, PsdΔ*alg8*::kan, which had a compromised Zn^2+^ accumulation, was not able to produce phenazines, even in the medium devoid of Zn^2+^. This pointed toward the importance of mineral nutrients in the antibiotic biosynthesis. Alternatively, this observation can be a result of a cross-talk between alginate that helps in better association with plant roots and antibiotic biosynthesis pathways.

A variety of PGP bacterial strains like *Azospirillum, Azotobacter, Bacillus, Pseudomonas*, and *Streptomyces* have earlier been implicated in biocontrol of plant pathogens like tomato mottle virus, tobacco necrosis virus, *Rhizoctonia bataticola*, and *Fusarium avenaceum* (Bhattacharyya and Jha, 2012 and references therein). In the present study, competence of Zn^2+^-laden biomass of strain Psd in biocontrol of plant pathogens, *F. oxysporum* and *F. graminearum* had a stimulatory effect on biocontrol potential, with up to 80% inhibition obtained in case of *F. oxysporum*. This finding was in line with the results obtained in tomato, wherein the disease suppression by *P. fluorescens* CHA0 was augmented upon addition of Zn^2+^ (Duffy and Défago, 1997). It was proposed that Zn^2+^ amendment abolished fusaric acid production by *F. oxyposrum*, reducing the pathogenicity of the fungus (Duffy and Défago, 1997). Application of Zn^2+^ alone or in combination with the biocontrol agent *P. aeruginosa* significantly decreased the penetration of the root knot nematode *Meloidogyne javanica* in tomato (Siddiqui et al., 2002). The reduced pathogenecity can also be attributed to increased production of antifungal metabolites. On the other hand, PsdΔ*alg8*::kan exhibited significantly low biocontrol activity. The strain displayed reduced biomass inhibition of *F. oxysporum* and was not able to inhibit *F. graminearum* at all. This observation corroborated inability of the mutant to produce/release phenazines.

The stimulatory effect of Fluorescent *Pseudomonas* strain Psd on root growth has been studied earlier (Kochar et al., 2011; Sirohi et al., 2015). It has been established that biofilm formation by PGP bacteria leads to improved plant growth by facilitating dense bacterial population to produce various phytohormones, antibiotics, beneficial secondary metabolites and exoenzymes (Danhorn and Fuqua, 2007; Koul et al., 2015). The fact that Zn^2+^ accumulation triggers an improved PGP response in strain Psd was also observed in terms of improved root growth and proliferation. Bacterial attachment led to increase in formation of root hairs, indicative of better root growth. The functional implication, on the other hand, of decreased EPS production and subsequently, inability to form biofilms by strain PsdΔ*alg8*::kan was reflected in the mutant's inability to lead to a significant improvement in plant growth.

Overall, as sets out in the present study, alginates not only mediate the Zn^2+^ biosorption by strain Psd, but may also serve as important determinants in plant-growth-promotion. Interestingly, a possible dependence of phenazine biosynthesis with that of alginate production is also indicated in the study. However, this part warrants further investigation. The study also ascertains the rhizosphere competence of the strain in heavy-metal contaminated soils, without jeopardizing their PGP potential.

## Author contributions

AU, MK, SS, and MR conceived and designed the experiments. AU and MK performed the experiments and analyzed the data. SS and MR contributed financial assistance. AU, MK, and MR wrote the paper.

### Conflict of interest statement

The authors declare that the research was conducted in the absence of any commercial or financial relationships that could be construed as a potential conflict of interest.

## Supplementary material

Figure S1 **Schematic representation of the alginate biosynthesis pathway operating in ***Pseudomonas*** sp**.

Figure S2 **Schematic representation of (A)** upstream and downstream region of *alg8* gene; **(B)** homologous recombination-based mechanism to replace *alg8* gene with *kan*^*r*^ cassette; **(C)** PCR amplification of five putative constructs (pBKS_*alg8*_up/kan/down) with *alg8* upstream, downstream and kanamycin resistance gene specific primers. M-1 kb DNA ladder (NEB, USA); Lanes 1–5 represent amplification of ~500 bp region upstream to *alg8*; Lanes 6–10 represent amplification of ~500 bp region downstream to *alg8* and; Lanes 11–15 represent amplification of the *kan*^r^ gene.

Figure S3 **Flow diagram of the protocol used for Zn^**2+**^ estimation in bacterial cells**.

Figure S4 **Quantitative determination of alginates in EPS secreted from strain Psd grown in different Zn^**2+**^ concentrations by DMMB-binding assay (^********^***p*** < 0.0001)**.

Figure S5 **Biofilm production by Psd and PsdΔ***alg8***::***kan*** in GMM supplemented with increasing Zn^**2+**^ concentrations (^*****^***P*** < 0.05, ^*******^***P*** < 0.001, ^********^***P*** < 0.0001, ns, not significant)**.

Figure S6 **Bioassay showing the effect of treatment of wheat seeds with (A)** *Pseudomonas* strain Psd, and **(B)** strain PsdΔ*alg8*::*kan*, on seedling growth after 6-days of inoculation.

Figure S7 **Scanning electron microscopy of wheat roots inoculated with ***Pseudomonas*** strain Psd grown at increasing Zn^**2+**^ concentrations to show the effect on roots proliferation: Untreated control (A)**, roots treated with wild type strain Psd grown in absence **(B)**, and presence of 2 mM Zn^2+^ **(C)**. RH, root hair. Scale bars vary from 20 to 100 μm and are shown in each image.

Table S1 **List of strains and plasmids used in the study**.
