# Supplementary figures and images for: Players over the Surface: Unraveling the Role of Exopolysaccharides in Zinc Biosorption by Fluorescent Pseudomonas Strain Psd
Source: Front Microbiol. 2017 Feb 24;8:284. doi: 10.3389/fmicb.2017.00284 (PMC5323414; doi:10.3389/fmicb.2017.00284)

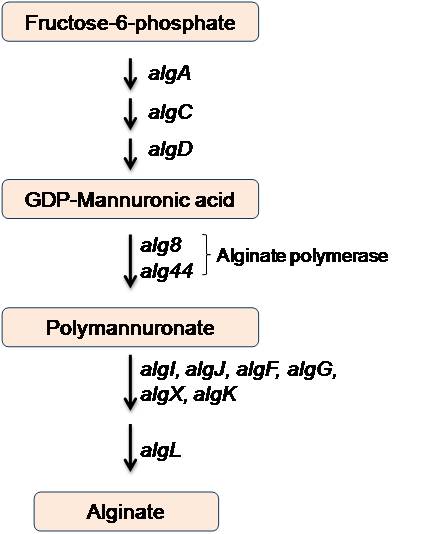

Supplement: Figure S1 — Schematic representation of the alginate biosynthesis pathway operating in Pseudomonas sp. [file Image1.JPEG]

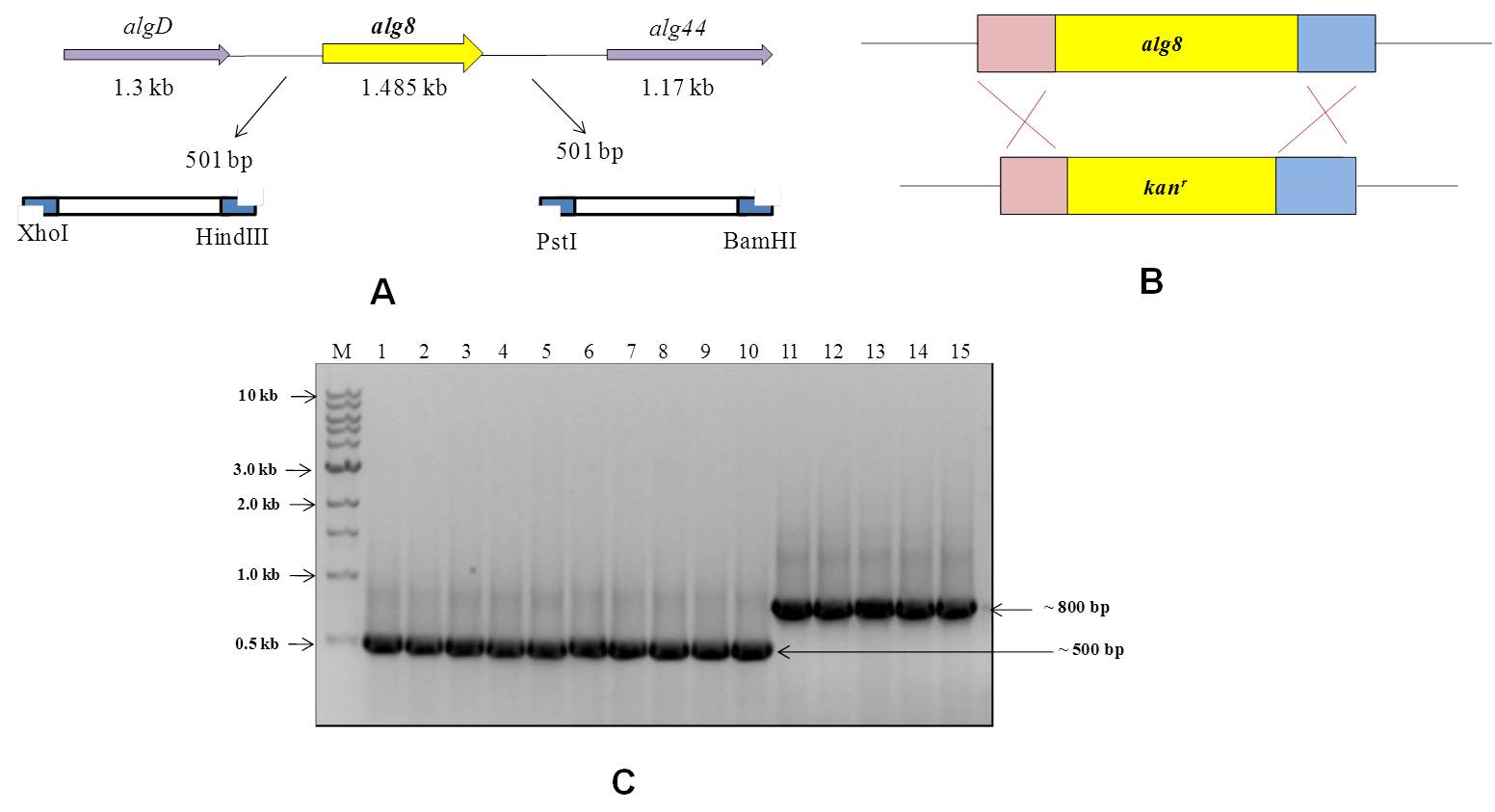

Supplement: Figure S2 — Schematic representation of (A) upstream and downstream region of alg8 gene; (B) homologous recombination-based mechanism to replace alg8 gene with kanr cassette; (C) PCR amplification of five putative constructs (pBKS_alg8_up/kan/down) with alg8 upstream, downstream and kanamycin resistance gene specific primers. M-1 kb DNA ladder (NEB, USA); Lanes 1–5 represent amplification of ~500 bp region upstream to alg8; Lanes 6–10 represent amplification of ~500 bp region downstream to alg8 and; Lanes 11–15 represent amplification of the kanr gene. [file Image2.JPEG]

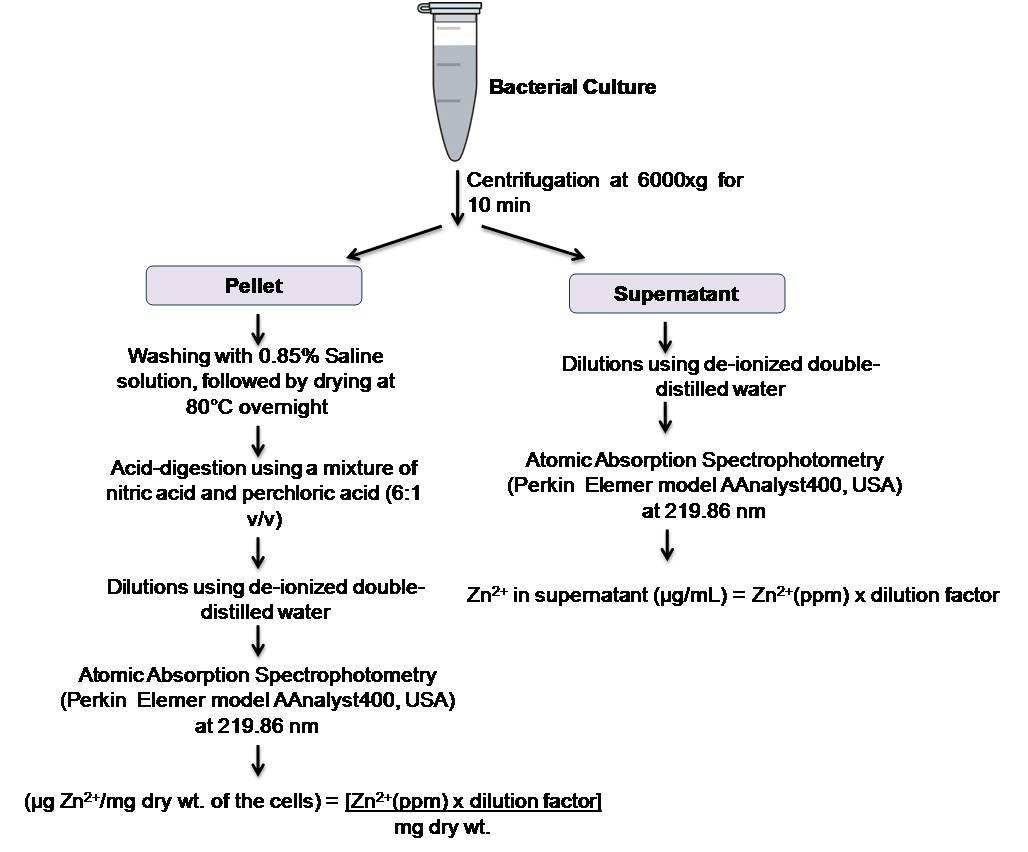

Supplement: Figure S3 — Flow diagram of the protocol used for Zn2+ estimation in bacterial cells. [file Image3.JPEG]

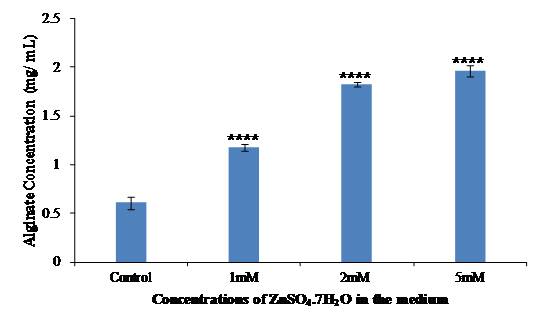

Supplement: Figure S4 — Quantitative determination of alginates in EPS secreted from strain Psd grown in different Zn2+ concentrations by DMMB-binding assay (****p < 0.0001). [file Image4.JPEG]

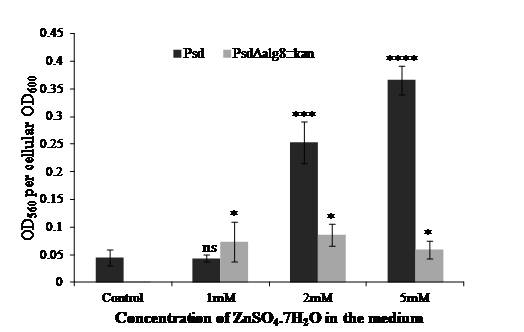

Supplement: Figure S5 — Biofilm production by Psd and PsdΔalg8::kan in GMM supplemented with increasing Zn2+ concentrations (*P < 0.05, ***P < 0.001, ****P < 0.0001, ns, not significant). [file Image5.JPEG]

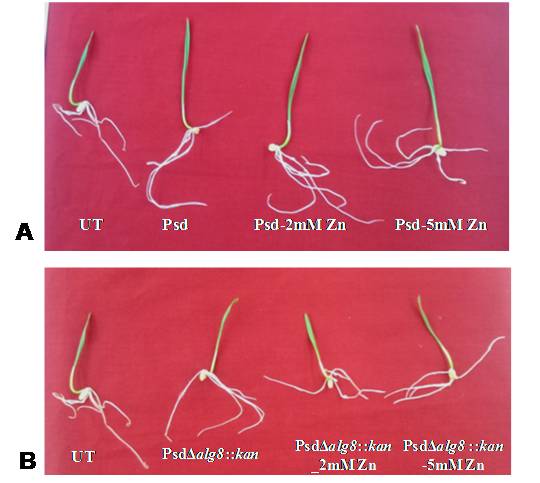

Supplement: Figure S6 — Bioassay showing the effect of treatment of wheat seeds with (A) Pseudomonas strain Psd, and (B) strain PsdΔalg8::kan, on seedling growth after 6-days of inoculation. [file Image6.JPEG]

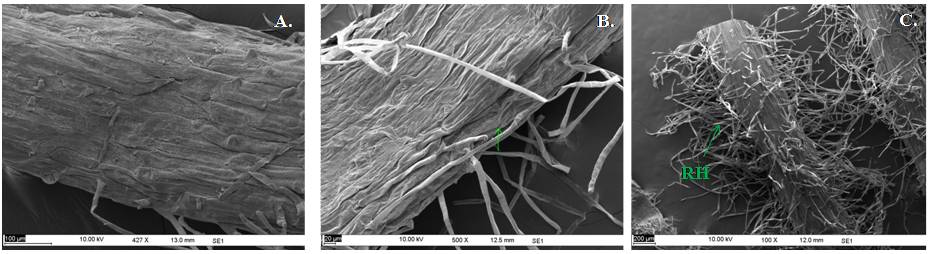

Supplement: Figure S7 — Scanning electron microscopy of wheat roots inoculated with Pseudomonas strain Psd grown at increasing Zn2+ concentrations to show the effect on roots proliferation: Untreated control (A), roots treated with wild type strain Psd grown in absence (B), and presence of 2 mM Zn2+ (C). RH, root hair. Scale bars vary from 20 to 100 μm and are shown in each image. [file Image7.JPEG]
