# Supplementary material for: Players over the Surface: Unraveling the Role of Exopolysaccharides in Zinc Biosorption by Fluorescent Pseudomonas Strain Psd
Source: Front Microbiol. 2017 Feb 24;8:284. doi: 10.3389/fmicb.2017.00284 (PMC5323414; doi:10.3389/fmicb.2017.00284)
Supplement: Table S1 — List of strains and plasmids used in the study. [file Table1.DOC]

**Table S1:** List of strains and plasmids used in the study

| **Strain or Plasmid** | **Relevant characteristics** | **Reference** |
| --- | --- | --- |
| **BACTERIAL STRAINS** | | |
| Fluorescent *Pseudomonas strain* Psd | Wild-type strain, isolated from *Vigna mungo* roots, Tc^r^, Ap^r^, Cm^r^ | Dr. A.K. Saxena, IARI, New Delhi, India |
| *Escherichia coli* strain XL1-Blue | *sup*E44 *hsd*R17 (r_k_^-^ m_k_^+^) *rec*A1 *end*A1 *thi*-1 *gyr*A96 *relA1 lac- F’[proAB+ lacIq lac Z Δ*D*M15 Tn10*(Tc^r^) | Lab Stock |
|  | **FUNGAL STRAINS** |  |
| *Fusarium oxysporum* | Standard plant pathogen causing damping off in tomato plants | Dept of Pathology, IARI, New Delhi |
| *Fusarium graminearum* (MTCC 1893) | Standard plant pathogen causing seedling blight in cereals | MTCC, Chandigarh |
|  | **PLASMIDS** |  |
| pBKS+ | Cloning vector; ColE1 replicon; Ap^r^ | Lab Stock |
| pGEM-T | Cloning vector; ColE1 replicon; Ap^r^ | Promega, USA |
| pBKS-*kan* | Cloning vector; ColE1 replicon; Ap^r^, Km^r^ | Lab Stock |
